# Adverse Drug Reactions Causing Admission to a Paediatric Hospital

**DOI:** 10.1371/journal.pone.0050127

**Published:** 2012-12-04

**Authors:** Ruairi M. Gallagher, Jennifer R. Mason, Kim A. Bird, Jamie J. Kirkham, Matthew Peak, Paula R. Williamson, Anthony J. Nunn, Mark A. Turner, Munir Pirmohamed, Rosalind L. Smyth

**Affiliations:** 1 Department of Women’s and Children’s Health, University of Liverpool, Liverpool, United Kingdom; 2 Research and Development, Alder Hey Children’s Hospital NHS Foundation Trust, Liverpool, United Kingdom; 3 Department of Biostatistics, University of Liverpool, Liverpool, United Kingdom; 4 National Institute for Health Research Medicines for Children Research Network, University of Liverpool, Alder Hey Hospital (previously: Department of Pharmacy, Alder Hey Children’s NHS Foundation Trust), Liverpool, United Kingdom; 5 Department of Pharmacology, University of Liverpool, Liverpool, United Kingdom; Nottingham University, United Kingdom

## Abstract

**Objective(s):**

To obtain reliable information about the incidence of adverse drug reactions, and identify potential areas where intervention may reduce the burden of ill-health.

**Design:**

Prospective observational study.

**Setting:**

A large tertiary children’s hospital providing general and specialty care in the UK.

**Participants:**

All acute paediatric admissions over a one year period.

**Main Exposure:**

Any medication taken in the two weeks prior to admission.

**Outcome Measures:**

Occurrence of adverse drug reaction.

**Results:**

240/8345 admissions in 178/6821 patients admitted acutely to a paediatric hospital were thought to be related to an adverse drug reaction, giving an estimated incidence of 2**.**9% (95% CI 2.5, 3.3), with the reaction directly causing, or contributing to the cause, of admission in 97.1% of cases. No deaths were attributable to an adverse drug reaction. 22.1% (95% CI 17%, 28%) of the reactions were either definitely or possibly avoidable. Prescriptions originating in the community accounted for 44/249 (17.7%) of adverse drug reactions, the remainder originating from hospital. 120/249 (48.2%) reactions resulted from treatment for malignancies. The drugs most commonly implicated in causing admissions were cytotoxic agents, corticosteroids, non-steroidal anti-inflammatory drugs, vaccines and immunosuppressants. The most common reactions were neutropenia, immunosuppression and thrombocytopenia.

**Conclusions:**

Adverse drug reactions in children are an important public health problem. Most of those serious enough to require hospital admission are due to hospital-based prescribing, of which just over a fifth may be avoidable. Strategies to reduce the burden of ill-health from adverse drug reactions causing admission are needed.

## Introduction

Children are vulnerable to adverse drug reactions (ADRs). [1], [2], [3], [4], [5], [6] Spontaneous reporting systems, such as the UK Yellow Card scheme, [7] are subject to under reporting of ADRs, even those which are severe. [8] To obtain reliable information about the incidence of ADRs prospective studies are needed. A systematic review of observational studies of ADRs causing paediatric hospital admissions, between 1976 to 1996, estimated the rate of ADR admissions to be 2.1% (95% CI 1.0, 3.8). [9] A further review of prospective paediatric studies published between 2001 and 2007 did not identify any large studies of the incidence and nature of ADRs causing hospital admission. [10]

Some results of the present study, prior to publication, were included in a recent systematic review by Smyth et al in 2011 [11]. The authors reviewed prospective studies researching ADRs in three settings; ADRs in-patients, those causing acute admission to hospital and those occurring in out-patients. Incidence rates for ADRs causing hospital admission ranged from 0.4% to 10.3% of all children (pooled estimate of 2.9% (2.6%, 3.1%)). Only 19/102 studies, from all three settings, assessed avoidability.

**Table 1:** Univariate analyses of ADRs by age.

Age (years, months)[Median; Q1, Q3]	All	No ADR	ADR	Mann–Whitney U	P–value
*All*	[3y 1m; 9m, 9y] (n = 4656)	[3y 0m; 9m, 9y] (n = 4514)	[6y 0m; 2y 4m, 11y] (n = 142)	244161	<0.001
*Oncology*	[6y; 3y 6m, 12y] (n = 74)	[6y; 3y 6m, 13y] (n = 33)	[6y; 3y 0m, 10y] (n = 41)	580.5	0.296
*Non–Oncology*	[3y; 9m, 9y] (n = 4582)	[2y 11m; 9m, 9y] (n = 4481)	[6y; 1y 7m, 11y] (n = 101)	178319.5	<0.001

The aim of this study was to prospectively identify ADRs in children causing hospital admission during a one year period in order to quantify the burden of ADRs and characterise their features. The investigators aimed to determine the avoidability of identified ADRs and detail the reasons for determining reactions as avoidable. This aspect of ADRs causing admission in children has not been fully addressed in previous studies.

**Table 2:** Univariate analyses of ADRs by number of medicines taken.

Drug Count [Median; Q1, Q3]	All	No ADR	ADR	Mann–Whitney U	P–value
*All*	[2]; [1], [3] (n = 4656)	[2]; [1], [3] (n = 4514)	[6]; [3], [9] (n = 142)	115391.5	<0.001
*Oncology*	[6]; [4], [9] (n = 74)	[4]; [3], [7] (n = 33)	[8]; [5], [10] (n = 41)	380.5	0.001
*Non–Oncology*	[2]; [1], [3] (n = 4582)	[2]; [1], [3] (n = 4481)	[5]; [3], [9] (n = 101)	100371.5	<0.001

## Methods

We prospectively screened all unplanned admissions to a tertiary paediatric hospital for ADRs over a one year period, including weekends and holidays, from 1^st^ July 2008 to 30^th^ June 2009. Admissions were excluded if they were planned, or occurred as a result of accidental or intentional overdose. Patients admitted to an Accident and Emergency (A&E) department short-stay ‘observation ward’ were not included. [12] The definition of ADR used was that of Edwards and Aronson which is “an appreciably harmful or unpleasant reaction, resulting from an intervention related to the use of a medicinal product, which predicts hazard from future administration and warrants prevention or specific treatment, or alteration of the dosage regimen, or withdrawal of the product.” [13] This definition was chosen as it describes only clinically significant adverse reactions that cause harm and it includes the concept of preventive action.

**Table 3:** Multivariate logistic regression analysis for risk factors for occurrence of ADR admission.

Parameter	Odds Ratio (OR)	95% CI for OR	P–value
Gender	0.77	0.52, 1.12	0.17
Age	1.04	1, 1.08	0.03
Oncology	29.71	17.35, 50.88	<0.01
Number of medicines	1.24	1.19, 1.29	<0.01

a Variable(s) entered on step 1: Gender (Male), Age, Oncology, Number of medicines.

Before the study began, an educational program was undertaken amongst clinicians of all grades to raise awareness about the importance of taking detailed medication histories. A structured medication history section was added to medical admission documentation to ensure details were taken about medication in the preceding two weeks. We identified all unplanned admissions in the previous 24 hours daily from hospital information systems. The study team collected the following information from case notes: age, sex, presenting complaint, clinical history, diagnosis (if available), and medications, including over-the-counter drugs, taken in the preceding two weeks. If any information was unclear, study team members interviewed the family to clarify the history.

**Table 4:** Classification of drugs associated with ADR admissions.

Drug class(No^.^ of cases)	No of drugs	Drugs	ADRs
Cytotoxics (110)	275	Vincristine 51, Doxorubicin 38, Methotrexate 35, Etoposide 30,Mercaptopurine 27, Cytarabine 24, Ifosfamide 18, Cyclophosphamide15, Carboplatin 7, Vinblastine 5, Pegasparaginase 5, Dactinomycin 5,Daunorubicin 4, Cisplatin 3, Irinotecan 3, Temozolomide 2,Fludarabine 1, Amsacrine 1, Imatinib 1	Neutropenia 89, Thrombocytopenia 55, Anaemia 38, Vomiting 8, Mucositis 8, Deranged Liver Function Tests 7, Immunosuppression 7, Diarrhoea 5, Nausea 4, Constipation 3, Headache 2, Abdominal pain 1, Back pain 1, Haematuria 1, Leukencephalopathy 1, Deranged renal function 1
Corticosteroids (102)	107	Dexamethasone 68, Prednisolone 33, Hydrocortisone 2,Betamethasone 1, Mometasone 1,Methylprednisolone 1, Fluticasone 1	Immunosuppression 71, Post–op bleeding 23, Hyperglycaemia 3, Hypertension 1, Gastritis 1, Increased appetite 1, Impaired healing 1, Adrenal suppression 1
NSAIDs (31)	43	Ibuprofen 28, Diclofenac 15	Post–op bleeding 27, Haematemesis 2, Constipation 1, Abdominal pain 1
Vaccines (22)	37	Diphtheria Tetanus Pertussis Inactivated polio HaemophilusInfluenza vaccine 11, Pneumococcal conjugate 9,Meningococcal C 8, MMR 7, Haemophilus Influenza B 1, Influenza 1	Fever 8, Rash 5, Irritability 4, Seizure 4, Vomiting 3, Pallor 1, Apnoea 1, Limb swelling 1, Lethargy 1, Thrombocytopenia 1, Diarrhoea 1, Abdominal pain 1, Respiratory distress 1, Kawasaki disease 1
Drugs affecting theimmune response (18)	26	Tacrolimus 15, Mycophenolate 7, Azathioprine 2,Methotrexate 1, Infliximab 1	Immunosuppression 18
Anti–bacterial (16)	17	Co–amoxiclav 4, Penicillin V 3, Amoxicillin 3, Flucloxacillin 2,Cefaclor 1, Cefalexin 1, Cefotaxime 1, Teicoplanin 1, Erythromycin 1	Diarrhoea 7, Rash 4, Vomiting 4, Lip swelling 1, Deranged LFTs 1, Thrush 1
Drugs used indiabetes (9)	13	Insulin detemir 4, Insulin aspart 3, Isophane insulin 2,Biphasic isophane 2, Human insulin 2,	Hypoglycaemia 9
Drugs used in status epilepticus (8)	12	Lorazepam 5, Diazepam 5, Midazolam 2	Respiratory depression 8
Opioid analgesia (6)	7	Dihydrocodeine 3, Codeine phosphate 3, Fentanyl 1	Constipation 4, Ileus 1, Decreased conscious level 1
Drugs used in nausea (4)	4	Ondansetron 4	Constipation 4
Anti–epileptic drugs (2)	2	Carbamazepine 1, Nitrazepam 1	Constipation 1, Respiratory depression 1
Drugs that suppress rheumatic disease (2)	2	Methotrexate 1, Anakinra 1	Immunosuppression 2
Other (16)	4	Calcium carbonate and Amlodipine 1, Oxybutynin 1, Baclofen 1	Constipation 3
	2	Dimeticone 1, Carbocysteine 1	Rash 2
	2	Desmopressin acetate 1, Alimemazine 1	Seizure 2
	10	Glucose and Dextrose 1, Propanolol 1, Acetazolomide 1,Spironolactone 1, Loperamide 1, Macrogols 1, Captopril 1,Alfacalcidol 1, Ethinylestradiol 1	Hyperglycaemia 1, Wheeze/Difficulty in breathing 1, Headache 1, Hyperkalaemia 1, Intestinal obstruction 1, Diarrhoea 1, Renal dysfunction 1, Hypercalcaemia 1, Inter–menstrual bleed 1

We cross-referenced presenting symptoms/signs against the medication history for each patient using the ADR profile for relevant drugs from the Summary of Product Characteristics (SPC) [14] in the Medicines Compendium or, if not available, the British National Formulary (BNF) [15]. We identified possible ADRs using this information combined with the clinical history and temporal relationships of the medication(s) taken. We reported all possible ADRs to the responsible clinicians and to the Yellow Card scheme.

**Figure 1:**
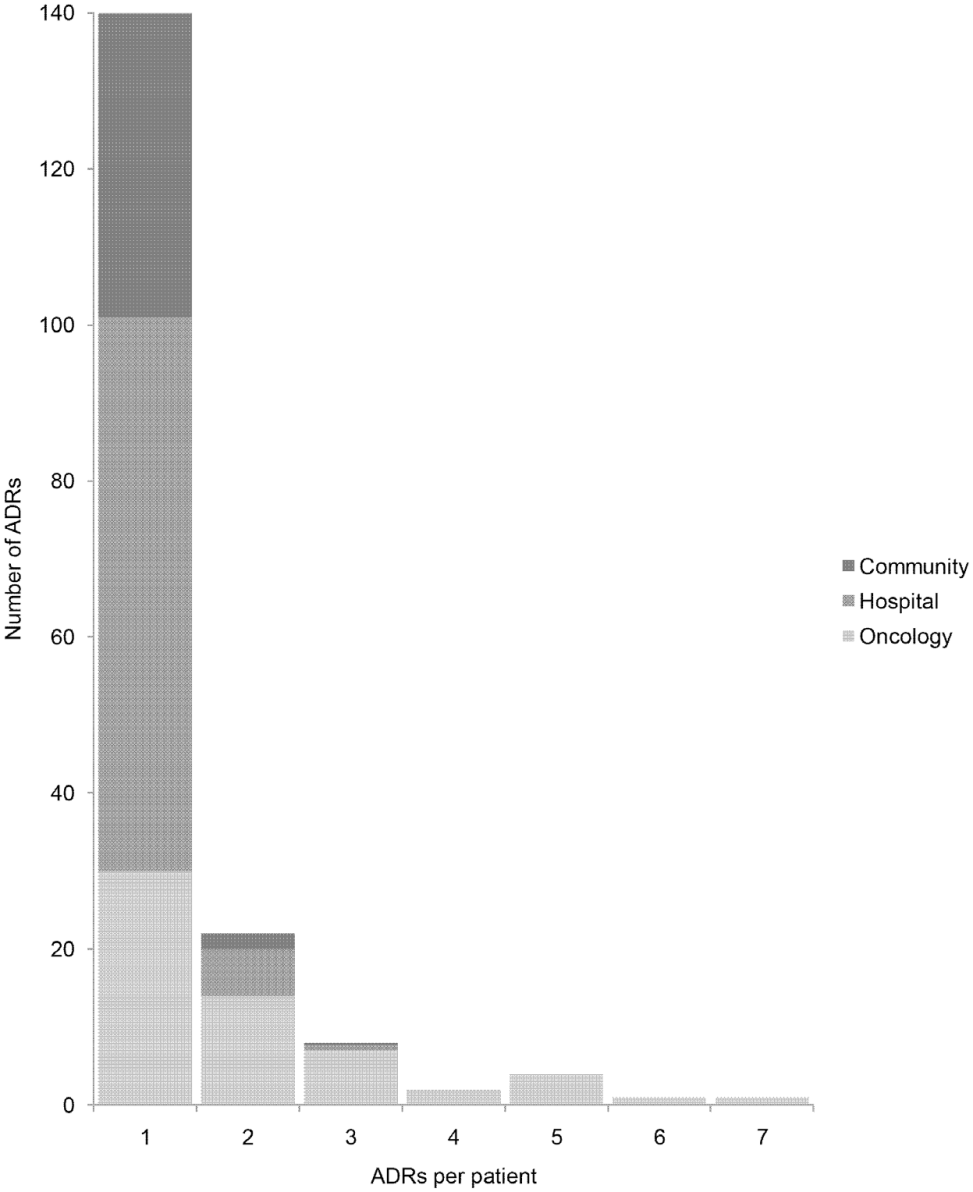
Number of ADRs per patient with ≥ one ADR according to origin of prescription.

We assessed the origin of prescription for drugs thought to be associated with ADRs using classifications of:

- *Community* – drugs where prescriptions originated in community settings, for example general practice, or where administration took place prior to hospital admission (e.g. paramedic administered)
- *Hospital* – drugs where the prescription originated, or administration took place, in hospital and then may or may not have been continued in community or outpatient settings
- *Oncology* – all drugs prescribed, or administered, from the oncology ward.

**Table 5:** Possibly and definitely avoidable cases and explanation of assessment result.

Avoidable	Frequency	ADR(s)	Drug Classes	Reason for potential avoidability
Definitely	3	Diarrhoea and/or vomiting	Anti-bacterial	Inappropriate indication, signs/symptoms of viral illness
Definitely	2	Constipation	Cytotoxics, Drugs used in nausea,Opioid analgesia	Appropriate prophylaxis not used
Definitely	1	Lip swelling, rash	Anti-bacterial	Same ADR previously to same medication
Definitely	1	Seizure	Antihistamine	Same ADR previously to similar medication
Definitely	1	Adrenal suppression	Corticosteroids	Avoidable with more rational prescribing (prolonged use of drugs) and improved monitoring
Definitely	1	Intestinal obstruction	Anti-motility drugs	Could be prevented by improved parent/patient education
Definitely	1	Deranged renal function	Drugs affecting the renin-angiotensin system	Avoidable with improved monitoring
Possibly	9	Hypoglycaemia	Drugs used in diabetes	Avoidable with improved patient education (e.g. insulin use when unwell) and more rational prescribing
Possibly	8	Respiratory depression	Drugs used in status epilepticus, Hypnotics	Alternative medicine available, Multiple doses given - avoidable with more rational prescribing
Possibly	6	Diarrhoea/vomiting	Anti-bacterial	Inappropriate indication, symptoms suggested viral infection
Possibly	5	Constipation	Antiepileptic drugs, Opioid analgesia,Drugs used in nausea, NSAIDs,Cytotoxics, Calcium-channel blockers,Calcium supplements	Prophylaxis not used
Possibly	4	Immunosuppression	Drugs affecting the immuneresponse, Corticosteroids	Possibly Avoidable with improved monitoring of drug levels, Avoidable with more rational prescribing
Possibly	2	Haematemesis	NSAIDs	Avoidable with improved patient education/more rational prescribing (less NSAID use)
Possibly	1	Neutropenia	Cytotoxics	Same ADR previously at same dose of medication
Possibly	1	Neutropenia,thrombocytopenia, anaemia	Cytotoxics	Superficial infection after recent admission with febrile neutropenia. Possibly avoidable by prolonging antibiotic use or commencing GCSF
Possibly	1	Hyperglycaemia	Corticosteroids	Avoidable with more rational prescribing (prolonged course steroids used)
Possibly	1	Hyperglycaemia	Parenteral preparations	Avoidable with more rational prescribing (more judicial use) or improved monitoring
Possibly	1	Seizure	Posterior pituitary hormones	Possibly inappropriate medication used for a patient with seizures
Possibly	1	Diarrhoea	Laxatives	Avoidable with improved patient education
Possibly	1	Ileus	Opioid analgesia	Avoidable with more rational prescribing (possibly use alternative analgesia)
Possibly	1	CNS depression	Opioid analgesia	Avoidable with improved patient education
Possibly	1	Vomiting	Cytotoxics	Possibly avoidable with more appropriate anti-emetic prophylaxis
Possibly	1	Gastritis	Corticosteroids	Previous gastritis. Possibly avoidable with improved prophylaxis
Possibly	1	Hypercalcaemia	Vitamins	Avoidable with improved monitoring

Drug class, according to BNF classification, was recorded for drugs implicated in causing ADRs. We performed assessment of causality using the Liverpool ADR Causality Assessment Tool, an algorithm developed by the investigators. [16] A novel aspect of the tool, which allows for a case to be classified as ‘definite’ causality, is that prior drug exposure that led to the same ADR was judged as being equivalent to a prospective re-challenge. Three investigators (RG, MT, AN) independently assessed causality for all ADR cases. Agreement on causality between all three investigators was taken as accepted consensus. Where the investigators did not achieve consensus, a fourth investigator (MuP) assessed cases to decide on causality.

The investigating group met to assess avoidability of the ADRs by consensus using the definitions developed by Hallas et al. [17] We determined the type of ADR (using the Rawlins and Thompson classification) [18] and severity using the Hartwig scale. [19] We chose these assessment tools to describe the ADRs in our study as they have been used in ADR studies by other investigators and can be completed quickly. Three investigators (AN, MuP, RLS) independently assessed 217/4514 (4.8%) reports of admissions exposed to medication, but deemed not to have had an ADR, to assess for occurrence of possible ADR cases wrongly classified by the study team.

We calculated the mean cost of ADR admissions to the study hospital, using information provided by the finance department for the cost of each case. Paediatric emergency admission data from the Health and Social Care Information Centre (National Health Service (NHS)), between 2009/2010, was used to estimate the total cost of ADR admissions annually in England.

### Ethics Statement

The Liverpool Paediatric Research Ethics Committee issued a formal opinion that this study was audit and informed consent from individual patients was not necessary.

### Statistical Analysis

Analyses of the rates of ADRs were based on the number of admissions with the rate expressed as ADR per 100 admissions, together with 95% confidence intervals. Other results are presented as medians and interquartile ranges or percentage frequencies and 95% percent confidence intervals. The formal statistical analysis was based on the data obtained at the first admission for patients exposed to a medication. Univariate statistical analyses were performed using the Mann-Whitney U test except for frequency data, which were analysed using a chi-square test. A multivariate logistic regression analysis was undertaken to calculate odds ratios for possible risk factors for ADR. A P-value <0.05 was regarded as being significant. All data were analyzed anonymously.

## Results

Over the study period, there were 6821 patients (3961 boys and 2860 girls) admitted 8345 times to the study hospital. The median number of admissions per patient was one, with 932 patients having more than one acute admission, up to a maximum of fifteen. 178 patients (94 boys, 84 girls) experienced 240 admissions with an ADR. This gives an incidence of 2.9 ADRs per 100 admissions (95% CI 2.5, 3.3). In 233 of 240 (97.1%) admissions an ADR was deemed to have directly caused, or contributed to, admission. There were 249 ADRs in 240 admissions, with nine admissions having two separate ADRs. 35/178 (19.7%) patients had more than one admission (maximum seven) with an ADR. Assessment of a sample of non-ADR cases (n = 217) confirmed that no admissions were due to ADRs.

There were 4656 patients exposed to medication in the two weeks prior to acute hospital admission. Of these, 142 (3%) had a suspected ADR on their first hospital admission. There was no significant difference between the proportion of boys (76/2677, 2.8%) and girls (66/1979, 3.3%) experiencing an ADR on their first admission, for the group as a whole or oncology patients studied separately. For non-oncology patients, there was a slightly higher proportion of girls admitted with an ADR (boys 48/2627 (1.8%), girls 53/1955 (2.7%), P = 0.044), although overall more boys than girls were admitted.

The median age of the 4656 patients who had been exposed to a drug on their first admission was 3 years 1 month (IQR 9 months, 9 years). Patients with an ADR (6y; 2y 4m, 11y) were significantly older (P<0.01) than those without (3y; 9m, 9y) (Table 1). There was no age difference between 41 oncology patients admitted with an ADR (6y; 3y, 10y) and 33 oncology patients admitted without an ADR (6y; 3y 6m, 13y). There was a significant age difference (P<0.01) between 101 non-oncology patients admitted with ADR (6y; 1y 7m, 11y) and 4481 admitted without ADR (2y 11m; 9m, 9y).

Patients admitted with an ADR had taken a greater number of drugs than those admitted for other reasons (Table 2). For patients admitted with an ADR (n = 142), the number of medicines taken was higher (6; 3, 9, P<0.001) than those for other reasons (n = 4514) (2; 1, 3). The number of medicines taken by oncology patients admitted with an ADR (8; 5, 10) was higher than those admitted without an ADR (4; 3, 7) and this difference was also found for non-oncology patients (with ADR 5; 3, 9: without ADR 2; 1, 3).

Logistic regression analysis showed a trend towards boys being less likely to experience an ADR than girls, with an odds ratio (OR) of 0.77 (95% CI 0.52, 1.12, P = 0.17) (Table 3). There was an increased likelihood of ADRs with increasing age (OR 1.04, 95% CI 1.003, 1.08, P = 0.03). No children were admitted with an ADR in the first month of life. Oncology patients were much more likely to have an ADR causing admission (OR 29.71, 95% CI 17.35, 50.88, P<0.001). The likelihood of a child being admitted with an ADR increased with the number of medicines taken (OR 1.24, 95% CI 1.19, 1.29, P<0.001). Therefore, for each additional medicine taken the risk of an ADR occurring increases by almost 25%.

### Drug Class

The main class of drugs contributing to ADR-related admissions (n = 110; 44.2%) was cytotoxic drugs (Table 4). Corticosteroids (n = 102, 41%), non-steroidal anti-inflammatory drugs (NSAIDs) (n = 31, 12.4%), vaccines (n = 22, 8.8%) and immunosuppressants (n = 18, 7.2%) were the next most commonly implicated drug classes causing ADR-related hospital admissions.

### ADRs

The most common ADRs were oncology related including neutropenia (89), thrombocytopenia (55) and anaemia (38). The next most common ADR was immunosuppression (74), occurring in both oncology and non-oncology patients. Post-operative bleeding, linked to peri-operative corticosteroid administration and/or NSAIDs, caused 28 admissions (26 post-tonsillectomy). Vomiting (15), diarrhoea (14), rash (11) and constipation (9) were all common ADRs causing admission. Hypoglycaemia in diabetic patients treated with regular insulin caused nine admissions. Respiratory depression following treatment for status epilepticus caused eight admissions to the hospital’s paediatric intensive care unit (PICU).

### Origin of Prescriptions

44/249 (17.7%) of ADRs were associated with prescriptions from the community, 85/249 (34.1%) with prescriptions originating in hospital for treatment of conditions other than oncology; 120/249 (48.2%) with prescriptions originating from oncology. Of the patients with one ADR (n = 140) in the study period, 39 (27.9%) occurred with community prescriptions, 71 (50.7%) with hospital prescriptions and 30 (21.4%) with oncology prescriptions; hospital-based prescriptions, particularly oncology, predominated in patients who had more than one ADR (Figure 1).

### ADR Assessments (Reaction Type, Causality, Severity, Avoidability)

238/249 (95.6%) ADRs were classified as type A (predictable from the known pharmacology) with 11/249 (4.4%) being type B (not predictable). Assessment of causality showed the majority of cases (94/249, 37.8%) to be in the ‘definite’ category. Oncology cases accounted for 80 of these 94 definite cases (Table S1). 92/238 (39.1%) type A reactions were assessed to be of definite causality. 8/11 (72.7%) type B reactions were assessed to be ‘possible.’

223/249 (89.6%) of the ADRs were classified as grade 3 (‘required treatment or drug administration discontinued’) according to the Hartwig severity scale, as we defined anyone requiring admission to hospital as ‘needing treatment.’ 14 (5.6%) were classified as grade 4 (‘resulted in patient transfer to higher level of care’) including respiratory depression (8), immunosuppression (4), neutropenia (1), fever/seizure (1) and leukencephalopathy (1). Three ADRs were classified as grade 5 (‘caused permanent harm or significant haemodynamic instability’). Two of these most severe ADRs occurred in oncology patients with febrile neutropenia and septicaemia and the remaining case was a child who required bowel resection for ileus following treatment with loperamide. No ADRs contributed to death. The majority (16/17, 94.1%) of the more severe reactions (≥ Grade 4 Hartwig severity score) were assessed to have definite or probable causality.

We determined 112/120 (93.3%) of the oncology patient admission ADRs to be unavoidable, with a further six being possibly avoidable and two definitely avoidable. These ‘definitely avoidable’ cases were oncology patients with constipation following treatment with vincristine and ondansetron (with one also having dihydrocodeine) without laxative prophylaxis.

Of the ADR admissions not associated with oncology patients, 82/129 ADRs (63.6%) were classified as unavoidable, 39 (30.2%) as possibly avoidable (14/39 prescribed from the community) and 8 (7.6%) as definitely avoidable (5/8 prescribed from the community). The eight ‘definitely avoidable’ comprised four patients prescribed antibiotics where the antibiotic choice or indication was deemed to be inconsistent with good practice, one patient with intestinal obstruction being treated with loperamide who had not passed stool for two days prior to admission, one patient who had a seizure after alimemazine having had two previous occurrences of seizure following anti-histamine use, one patient with deranged renal function which improved after cessation of captopril where improved renal function monitoring may have avoided the ADR, and one patient who presented with adrenal suppression following two years of continuous treatment with intranasal corticosteroids. The possibly avoidable cases and the reasons for their allocation are summarised in Table 5. 41/55 (74.5%) of possibly or definitely avoidable cases were classified as ‘definite’ or ‘probable’ causality.

### Cost of ADRs

We calculated the mean cost of 238/240 ADR admissions to the study hospital, using information provided by the finance department, to be £4753 per admission (95% CI £3439, £6066). Cost data were missing for two ADR admissions. Data from the Health and Social Care Information Centre (National Health Service (NHS)) [20] showed, in one year between 2009/2010, the total number of paediatric emergency admissions in England was approximately 597,800 (includes paediatrics and paediatric surgery, cardiology and neurology). We estimate the annual mean cost of ADR admissions to the NHS in England to be £82.4M. Using the upper and lower confidence intervals for our estimate of ADR incidence (2.5%, 3.3%), and study hospital costs, we estimate the cost to the NHS in England to be between £51.4–119.7M.

## Discussion

This prospective observational study is the largest of its kind in children and the only one to comprehensively assess causality, type of reaction, severity, avoidability and risk factors. In our setting, the majority of admissions associated with ADRs in children occurred as a result of prescriptions originating in hospital. Potential preventative strategies for ADRs causing admission in children should therefore be targeted at hospital prescribing. Our analysis of the ‘definitely avoidable’ ADRs in our study suggests careful attention to practical aspects of care, such as improved monitoring, following prescribing guidelines, patient education, and heightened suspicion about potential reactions could lead to a reduction in the frequency of this important problem.

This study gave an estimated ADR admission incidence of 2.9% (95% CI 2.5, 3.3), which is similar to a pooled estimate of 2.9% (2.6%, 3.1%) from a recent comprehensive systematic review. [11] The incidence of ADRs in this study was significantly less than that of a large US study published in 1988 (3.96%, 95%CI 3.52, 4.43). [2] In that study the top three drugs causing ADRs were phenobarbital, aspirin and phenytoin, all of which are used in children much less now because of safety concerns and because better alternatives are available. The majority of ADRs that were seen during our study were oncology related. Oncology patients are often exposed to medications causing ADRs, including neutropenia, nausea, vomiting, diarrhoea and thrombocytopenia, all of which may require admission. [21] These ADRs are expected and may be unavoidable given the underlying illness and the treatment options available. Although several studies have evaluated a potential preventative strategy for neutropenia [22], no definite evidence exists regarding the use of granulocyte-colony stimulating factors (GCSF) to prevent such ADRs [23].

Steroids, along with other immunosuppressants, increase risk of infection. [24] These ADR admissions were children taking steroids, admitted with proven bacterial, or viral infections associated with immunosuppression, such as shingles. Although such infections occur in healthy children, immunosuppressive therapy may be causal and this may be an under-recognised ADR.

The majority of admissions for post-operative bleeding (23/28) occurred in patients exposed to intravenous dexamethasone for anti-emetic prophylaxis, and non-steroidal anti-inflammatory drugs (NSAIDs). A few patients received either steroid or NSAIDs. Dexamethasone has been linked to post-tonsillectomy bleeding [25] but its role, and the role of NSAIDs, in causing secondary haemorrhage is yet to be determined. [26], [27] However, intra-operative steroids have played a major role in improving post-operative nausea and vomiting in children. [26], [28]

Respiratory depression following treatment of seizures with benzodiazepines, a well recognised event, [29] was the cause of eight PICU admissions, some of whom were transfers from other district general hospitals. Some occurred as a result of rectal diazepam used by paramedics in out-of-hospital care. The benefit/risk ratio of drugs used to treat seizures has been the objective of a number of clinical studies [30], [31], and there may be better drugs to treat seizures in children. [32]

Assessment of avoidability was undertaken by consensus approach using the definitions by Hallas. The definitions, which are based on avoidability linked to standards of care, are wide and may lead to variability in assessor rating. The Hallas criteria are less prescriptive than some other avoidability tools but there is little evidence to suggest preference for the use of any one avoidability tool. [33]

While this study has highlighted important ADRs, we cannot be certain of the aetiological fraction (the risk of an event occurring in the presence of a risk factor) for some of the drugs in their contribution to the ADRs. Further prospective, cohort studies that capture benefits and harms using validated tools and all medication exposures with adequate sample size are needed to assess this accurately and to estimate more precisely risks compared to benefits.

We calculated the cost of ADRs to the NHS in England using knowledge of the cost of admissions to the study hospital, our estimate of the incidence of ADRs causing admission and an estimate of total paediatric admissions annually to hospitals in England, although this may be an underestimate, as the multiplier which we used (total paediatric emergency admissions), did not include admissions of children from other specialities.

### Conclusion

We have demonstrated that ADRs cause a small but substantial proportion of admissions to hospital and some of these are serious and potentially avoidable. The results of this study should be used to inform paediatric pharmacovigilance practice. Preventing avoidable ADRs will require careful attention to good prescribing practice.

## Supporting Information

Table S1 Origin of prescription of ADR drugs by type of reaction, severity score, avoidability and causality assessment.
(DOCX)
