# Supplementary material for: Adverse Drug Reactions Causing Admission to a Paediatric Hospital
Source: PLoS One. 2012 Dec 4;7(12):e50127. doi: 10.1371/journal.pone.0050127 (PMC3514275; doi:10.1371/journal.pone.0050127)
Supplement: Table S1 — Origin of prescription of ADR drugs by type of reaction, severity score, avoidability and causality assessment. (DOCX) [file pone.0050127.s001.docx]

Table S1 Origin of prescription of ADR drugs by type of reaction, severity score, avoidability and causality assessment

|  | **Type of reaction** | | **Severity Score** | | | | | **Avoidability** | | | **Causality** | | |
| --- | --- | --- | --- | --- | --- | --- | --- | --- | --- | --- | --- | --- | --- |
|  | *A* | *B* | *1* | *2* | *3* | *4* | *5* | *Unavoidable* | *Possibly* | *Definitely* | *Possible* | *Probable* | *Definite* |
| **Oncology (120)** | 119 | 1 | 5 | 0 | 111 | 2 | 2 | 112 | 6 | 2 | 9 | 31 | 80 |
| **Hospital (85)** | 85 | 0 | 1 | 2 | 74 | 8 | 0 | 57 | 25 | 3 | 51 | 24 | 10 |
| **Community (44)** | 34 | 10 | 1 | 0 | 38 | 4 | 1 | 25 | 14 | 5 | 23 | 17 | 4 |
